# Tris[4,4′-(ethene-1,2-di­yl)dipyridinium] deca­vanadate dihydrate

**DOI:** 10.1107/S1600536810006215

**Published:** 2010-02-24

**Authors:** Roberto Fernandez de Luis, M. Karmele Urtiaga, José Luis Mesa, María I. Arriortua

**Affiliations:** a Dpto de Mineralogía y Petrología, Facultad de Ciencia y Tecnología, Universidad del País Vasco/E.H.U., PO Box 644, 48080 Bilbao, Spain; b Dpto Química Inorgánica, Facultad de Ciencia y Tecnología, Universidad del País Vasco/E.H.U., PO Box 644, 48080 Bilbao, Spain

## Abstract

The asymmetric unit of the title compound, (C_12_H_12_N_2_)_3_[V_10_O_28_]·2H_2_O, contains one half of a deca­vanadate anion, one and a half *trans*-1,2-bis­(4-pyridinio)ethene cations and one water mol­ecule. The V_10_O_28_ groups are involved in a three-dimensional hydrogen-bonding network through Ow—H⋯O, N—H⋯O and C—H⋯O inter­actions.

## Related literature

For general background to inorganic–organic vanadates, see: Zavalij & Whittingham (1999 ▶); Fernández de Luis *et al.* (2009*a* ▶). For inorganic–organic vanadates constructed from pyridyl ligands, see: Fernández de Luis *et al.* (2009*b* ▶); Khan *et al.* (2004 ▶); Zheng *et al.* (2001 ▶). For general background to deca­vanadates, see: Pope & Müller (1991 ▶, 1994 ▶); Rhule *et al.* (1998 ▶). For hydrogen bonding, see: Steiner (2002 ▶). 
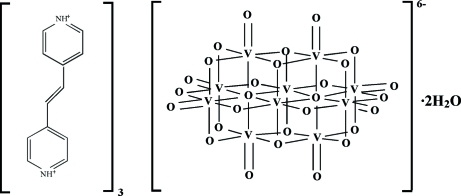


## Experimental

### 

#### Crystal data

(C_12_H_12_N_2_)_3_[V_10_O_28_]·2H_2_O
*M* *_r_* = 1546.14
Triclinic, 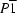

*a* = 9.7343 (4) Å
*b* = 11.7754 (5) Å
*c* = 12.2311 (5) Å
α = 113.072 (4)°
β = 105.396 (4)°
γ = 93.171 (3)°
*V* = 1223.33 (9) Å^3^
*Z* = 1
Mo *K*α radiation
μ = 1.92 mm^−1^
*T* = 293 K
0.18 × 0.14 × 0.08 mm

#### Data collection

Oxford Xcalibur2 diffractometer
Absorption correction: analytical (*CrysAlis RED*; Oxford Diffraction, 2008 ▶) *T*_min_ = 0.780, *T*_max_ = 0.887
10137 measured reflections
5498 independent reflections
3829 reflections with *I* > 2σ(*I*)
*R*_int_ = 0.029
3 standard reflections every 50 reflections  intensity decay: none

#### Refinement

*R*[*F* ^2^ > 2σ(*F* ^2^)] = 0.032
*wR*(*F* ^2^) = 0.076
*S* = 0.90
5498 reflections
378 parameters
2 restraints
H atoms treated by a mixture of independent and constrained refinement
Δρ_max_ = 0.64 e Å^−3^
Δρ_min_ = −0.39 e Å^−3^

### 

Data collection: *CrysAlis CCD* (Oxford Diffraction, 2008 ▶); cell refinement: *CrysAlis RED* (Oxford Diffraction, 2008 ▶); data reduction: *CrysAlis RED*; program(s) used to solve structure: *SIR92* (Altomare *et al.*, 1993 ▶); program(s) used to refine structure: *SHELXL97* (Sheldrick, 2008 ▶); molecular graphics: *PLATON* (Spek, 2009 ▶); software used to prepare material for publication: *TOPOS* (Blatov, 2006 ▶).

## Supplementary Material

Crystal structure: contains datablocks global, I. DOI: 10.1107/S1600536810006215/bg2319sup1.cif

Structure factors: contains datablocks I. DOI: 10.1107/S1600536810006215/bg2319Isup2.hkl

Additional supplementary materials:  crystallographic information; 3D view; checkCIF report

## Figures and Tables

**Table 1:** Hydrogen-bond geometry (Å, °)

*D*—H⋯*A*	*D*—H	H⋯*A*	*D*⋯*A*	*D*—H⋯*A*
O1w—H19⋯O11^i^	0.92 (3)	2.01 (4)	2.919 (3)	168 (4)
O1w—H20⋯O12	0.92 (3)	2.15 (3)	3.028 (3)	160 (3)
N1—H21⋯O9	0.86	1.88	2.721 (3)	164
N2—H22⋯O2^ii^	0.86	1.75	2.568 (3)	159
N3—H23⋯O5	0.86	1.71	2.564 (3)	172
C2—H2⋯O1^iii^	0.93	2.50	3.126 (4)	124
C4—H4⋯O1^iv^	0.93	2.32	3.107 (4)	143
C8—H8⋯O10	0.93	2.57	3.162 (4)	122
C11—H11⋯O3^iv^	0.93	2.33	3.254 (4)	173
C12—H12⋯O1w	0.93	2.57	3.230 (4)	128
C14—H14⋯O6^v^	0.93	2.54	3.202 (3)	128
C14—H14⋯O12^v^	0.93	2.39	3.293 (3)	162
C16—H16⋯O13^iv^	0.93	2.58	3.401 (4)	147
C17—H17⋯O13	0.93	2.60	3.187 (4)	122

Symmetry codes: (i) 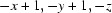; (ii) 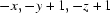; (iii) 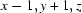; (iv) 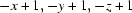; (v) 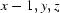.

**Fig. 1.**
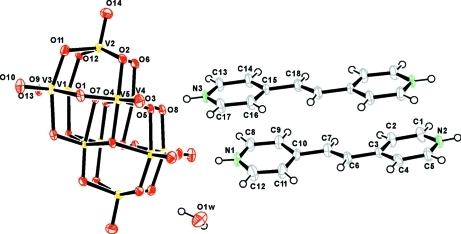
The molecular structure of the title compound showing the atom-labeling scheme.Displacement ellipsoids are shown at the 50% probability level.

**Fig. 2.**
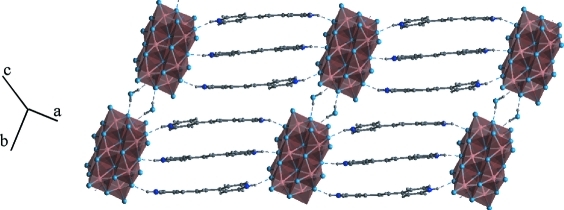
[V10O28]6- anions hydrogen bonded trough the water molecules and organic cations giving rise to sheets.

**Fig. 3.**
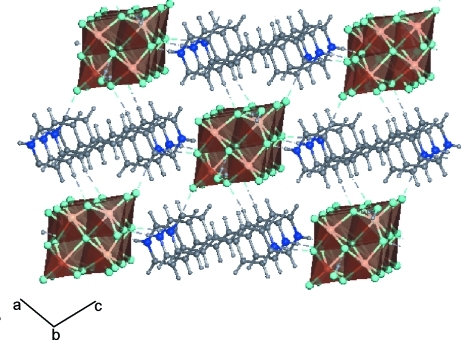
The crystal packing of (I). The sheets are stacked along the [101] direction.

## supplementary crystallographic information

### Comment

The title compound was synthesized as part of our studies focused on the construction of new inorganic-organic vanadates (Zavalij & Whittingham, 1999) with first row transition metal centres (Fernández *et al.*, 2009*b*). The pyridyl ligands have very often used since they have two nitrogen atoms that can bridge metal atoms to form polymeric compounds (Fernández *et al.*, 2009*a*), (Khan *et al.*, 2004), (Zheng *et al.*, 2001). However, the hydrothermal synthesis of the Co/Bpe/V*_x_*O*_y_* system give rise to the crystallization of the title compound as a minor product of the reaction.

The polyoxovanadates exhibit interesting physical and chemical properties with relevance to catalysis, biochemical processes (enzyme inhibitor or activator), medicine, and material science (Pope & Muller, 1991, 1994; Rhule *et al.*, 1998).

The asymmetric unit of (I) consists of one-half decavanadate anion, one and a half pyridinium cations and one water molecule. The decavanadate anion is composed of ten VO_6_ octahedra combined *via* shared edges and corners. Six octahedra are arranged in a 2 *x* 3 equatorial plane sharing edges; the other four octahedra are distributed above and below the equatorial plane, connected by shared sloping edges with the central six octahedra (Fig. 1).

The V—O bond lengths are classified according to the coordinative conditions of the oxygen atoms: terminal O atoms (V═O, 1.597 (2) - 1.608 (2) Å); double-bridging O atoms (V—O, 1.684 (2) - 2.083 (2) Å), triply bridging O atoms located on the surface of the [V_10_O_28_]^6-^ cluster (V—O, 1.968 (2) - 2.061 (2) Å), and one six-coordinate O atom (V—O 2.085 (2) - 2.344 (2) Å).

The supramolecular structure is formed by Ow—H···O, N—H···O and C—H···O hydrogen bonds, between the anion and the water molecules, between the cation and the anion, and between cation and water molecule (Steiner, 2002).

The decavanadate anions are hydrogen bonded through two water molecules (Fig.2) forming chains in the [010] direction (Fig. 1). The N—H groups of the organic cations interact with the surface oxygen atoms of the decavanadate anion (N1—H21···O9, N2—H22···O2, N3—H23···O5). Each V_10_O_28_ anion is linked trough three organic cations to other two V_10_O_28_ anion (Fig. 2). The hydrogen bonding trough the water molecules (O—H···O) and organic cations (N—H···O) of the [V_10_O_28_] clusters, generates the layers shown in the figure 2. The layers are stacked along the [101] direction *via* C—H···O hydrogen bonds, establishing the three dimensional supramolecular network (Fig. 3).

### Experimental

A mixture consisting of NaVO_3_ (0.135 mmol), 1,2-di(4-pyridyl)ethylene (0.135 mmol), Co(NO_3_)_2_.6H_2_O (0.135 mmol), and H_2_O (30 ml) in the molar ratio 1:1:1 was placed in a 50-ml Parr Teflon-lined autoclave. The initial pH value was adjusted to 4.0 with a 1*M* HNO_3_ solution under a vigorous stirring. The autoclave was sealed and heated for 3 days at 120°C. After the reaction a mixture of dark brown polycrystalline powder with a minor percentage of the title compound orange single crystals were obtained. In order to obtain the title compound as a single phase, the Co(NO_3_)_2_.6H_2_O was suppressed from the initial reactants. However, all attempts to obtain the title compound as a single phase after the hydrothermal reaction have been unsuccessful.

### Refinement

The H atoms belonging to the organic ligand were placed at geometrically idealized positions (C—H = 0.93 Å and N—H = 0.86 Å) and constrained to ride on their parent atoms [*U*_iso_(H)= 1.2*U*_eq_(C,*N*)]. H atoms of the water molecule were located in a difference map and refined with O—H inter-atomic distances restrained to 0.93 Å, with the standard deviation set at 0.01 Å.

### Crystal data

**Table:**

(C_12_H_12_N_2_)_3_[V_10_O_28_]·2H_2_O	*Z* = 1
*M**_r_* = 1546.14	*F*(000) = 768
Triclinic, *P*1	*D*_x_ = 2.099 Mg m^−^^3^
Hall symbol: -P 1	Mo *K*α radiation, λ = 0.71073 Å
*a* = 9.7343 (4) Å	Cell parameters from 4312 reflections
*b* = 11.7754 (5) Å	θ = 2.6–28.9°
*c* = 12.2311 (5) Å	µ = 1.92 mm^−^^1^
α = 113.072 (4)°	*T* = 293 K
β = 105.396 (4)°	Plate, orange
γ = 93.171 (3)°	0.18 × 0.14 × 0.08 mm
*V* = 1223.33 (9) Å^3^	

### Data collection

**Table:**

Oxford Xcalibur2 diffractometer	3829 reflections with *I* > 2σ(*I*)
Radiation source: fine-focus sealed X-ray tube	*R*_int_ = 0.029
graphite	θ_max_ = 29.0°, θ_min_ = 2.7°
profile data from q/2q scans	*h* = −9→12
Absorption correction: analytical (*CrysAlis RED*; Oxford Diffraction, 2008)	*k* = −14→12
*T*_min_ = 0.780, *T*_max_ = 0.887	*l* = −15→15
10137 measured reflections	3 standard reflections every 50 reflections
5498 independent reflections	intensity decay: none

### Refinement

**Table:**

Refinement on *F*^2^	2 restraints
Least-squares matrix: full	H atoms treated by a mixture of independent and constrained refinement
*R*[*F*^2^ > 2σ(*F*^2^)] = 0.032	*w* = 1/[σ^2^(*F*_o_^2^) + (0.0377*P*)^2^] where *P* = (*F*_o_^2^ + 2*F*_c_^2^)/3
*wR*(*F*^2^) = 0.076	(Δ/σ)_max_ < 0.001
*S* = 0.90	Δρ_max_ = 0.64 e Å^−^^3^
5498 reflections	Δρ_min_ = −0.39 e Å^−^^3^
378 parameters	

### Special details

**Table:**

Geometry. All e.s.d.'s (except the e.s.d. in the dihedral angle between two l.s. planes) are estimated using the full covariance matrix. The cell e.s.d.'s are taken into account individually in the estimation of e.s.d.'s in distances, angles and torsion angles; correlations between e.s.d.'s in cell parameters are only used when they are defined by crystal symmetry. An approximate (isotropic) treatment of cell e.s.d.'s is used for estimating e.s.d.'s involving l.s. planes.

### Fractional atomic coordinates and isotropic or equivalent isotropic displacement parameters (Å^2^)

**Table:**

	*x*	*y*	*z*	*U*_iso_*/*U*_eq_	
O1W	0.6206 (3)	0.5760 (2)	0.2406 (2)	0.0481 (6)	
V1	0.56372 (4)	0.24085 (4)	0.17922 (4)	0.01678 (11)	
V4	0.67454 (4)	0.01106 (4)	0.01759 (4)	0.01442 (10)	
V5	0.55805 (4)	0.00281 (4)	0.23104 (4)	0.01684 (11)	
V2	0.66272 (5)	0.24385 (4)	−0.04145 (4)	0.01972 (11)	
V3	0.34102 (5)	0.22893 (4)	−0.06201 (4)	0.02019 (11)	
O6	0.78126 (17)	0.11594 (15)	0.00005 (15)	0.0193 (4)	
O3	0.58831 (19)	0.08980 (17)	0.37727 (15)	0.0251 (4)	
O8	0.77818 (17)	−0.09058 (15)	0.04656 (15)	0.0187 (4)	
O13	0.59849 (19)	0.32733 (16)	0.32570 (15)	0.0259 (4)	
O9	0.40551 (18)	0.30068 (15)	0.10994 (15)	0.0198 (4)	
O5	0.41516 (17)	0.09802 (15)	0.16167 (14)	0.0152 (4)	
O10	0.2165 (2)	0.30576 (18)	−0.09039 (17)	0.0311 (5)	
O12	0.68888 (18)	0.31452 (15)	0.13329 (15)	0.0203 (4)	
O2	0.39991 (18)	−0.11428 (15)	0.20214 (15)	0.0192 (4)	
O11	0.50190 (19)	0.31199 (16)	−0.07012 (15)	0.0219 (4)	
O7	0.50263 (17)	0.10133 (14)	−0.02292 (14)	0.0156 (4)	
O4	0.67716 (17)	0.10895 (15)	0.18500 (14)	0.0162 (4)	
O1	0.68157 (18)	−0.10149 (16)	0.22455 (15)	0.0203 (4)	
O14	0.7885 (2)	0.32950 (18)	−0.05078 (17)	0.0311 (5)	
N3	0.2433 (2)	0.2213 (2)	0.2692 (2)	0.0243 (5)	
H23	0.2959	0.1735	0.2323	0.029*	
N1	0.2556 (3)	0.4870 (2)	0.1955 (2)	0.0313 (6)	
H21	0.3068	0.4358	0.1604	0.038*	
N2	−0.2323 (3)	1.0304 (2)	0.6653 (2)	0.0301 (6)	
H22	−0.2872	1.0732	0.7040	0.036*	
C9	0.0305 (3)	0.5473 (3)	0.1930 (2)	0.0310 (7)	
H9	−0.0682	0.5339	0.1520	0.037*	
C3	−0.0588 (3)	0.8902 (2)	0.5429 (2)	0.0230 (6)	
C15	0.0758 (3)	0.3752 (2)	0.3866 (2)	0.0230 (6)	
C7	−0.0022 (3)	0.7264 (3)	0.3679 (3)	0.0299 (7)	
H7	−0.0979	0.7137	0.3193	0.036*	
C17	0.3018 (3)	0.3073 (3)	0.3866 (2)	0.0316 (7)	
H17	0.3992	0.3141	0.4277	0.038*	
C2	−0.2037 (3)	0.8813 (3)	0.4807 (3)	0.0317 (7)	
H2	−0.2438	0.8275	0.3963	0.038*	
C14	0.0182 (3)	0.2840 (3)	0.2653 (2)	0.0301 (7)	
H14	−0.0792	0.2735	0.2218	0.036*	
C10	0.0919 (3)	0.6475 (2)	0.3090 (2)	0.0252 (6)	
C6	0.0367 (3)	0.8142 (3)	0.4839 (2)	0.0273 (6)	
H6	0.1330	0.8287	0.5319	0.033*	
C8	0.1146 (3)	0.4683 (3)	0.1390 (3)	0.0324 (7)	
H8	0.0725	0.4006	0.0616	0.039*	
C4	−0.0052 (3)	0.9733 (3)	0.6680 (3)	0.0330 (7)	
H4	0.0914	0.9819	0.7124	0.040*	
C16	0.2216 (3)	0.3861 (3)	0.4479 (3)	0.0325 (7)	
H16	0.2643	0.4462	0.5296	0.039*	
C11	0.2410 (3)	0.6652 (3)	0.3648 (3)	0.0383 (8)	
H11	0.2863	0.7323	0.4419	0.046*	
C18	−0.0196 (3)	0.4549 (2)	0.4436 (2)	0.0280 (6)	
H18	−0.1159	0.4400	0.3953	0.034*	
C13	0.1052 (3)	0.2090 (3)	0.2092 (3)	0.0308 (7)	
H13	0.0660	0.1483	0.1273	0.037*	
C5	−0.0935 (3)	1.0428 (3)	0.7266 (3)	0.0369 (8)	
H5	−0.0560	1.0994	0.8103	0.044*	
C1	−0.2869 (3)	0.9529 (3)	0.5454 (3)	0.0346 (7)	
H1	−0.3840	0.9467	0.5039	0.041*	
C12	0.3201 (3)	0.5841 (3)	0.3063 (3)	0.0427 (8)	
H12	0.4195	0.5963	0.3436	0.051*	
H19	0.580 (4)	0.600 (4)	0.178 (3)	0.103 (17)*	
H20	0.629 (4)	0.4968 (18)	0.190 (3)	0.090 (15)*	

### Atomic displacement parameters (Å^2^)

**Table:**

	*U*^11^	*U*^22^	*U*^33^	*U*^12^	*U*^13^	*U*^23^
O1W	0.0659 (17)	0.0344 (15)	0.0435 (14)	0.0091 (12)	0.0161 (13)	0.0166 (12)
V1	0.0169 (2)	0.0135 (2)	0.0151 (2)	0.00251 (18)	0.00647 (17)	0.00015 (18)
V4	0.0112 (2)	0.0148 (2)	0.0138 (2)	0.00369 (17)	0.00515 (17)	0.00151 (17)
V5	0.0167 (2)	0.0183 (2)	0.0130 (2)	0.00517 (18)	0.00559 (17)	0.00319 (18)
V2	0.0200 (2)	0.0171 (2)	0.0202 (2)	0.00216 (18)	0.00929 (18)	0.00423 (19)
V3	0.0209 (2)	0.0195 (2)	0.0203 (2)	0.00956 (19)	0.00864 (19)	0.00638 (19)
O6	0.0153 (9)	0.0184 (10)	0.0199 (9)	0.0014 (7)	0.0075 (7)	0.0025 (8)
O3	0.0279 (11)	0.0268 (11)	0.0150 (9)	0.0061 (8)	0.0077 (8)	0.0024 (8)
O8	0.0152 (9)	0.0212 (10)	0.0185 (9)	0.0071 (7)	0.0073 (7)	0.0053 (8)
O13	0.0291 (11)	0.0213 (10)	0.0185 (9)	0.0018 (8)	0.0088 (8)	−0.0010 (8)
O9	0.0214 (10)	0.0160 (9)	0.0201 (9)	0.0079 (7)	0.0103 (7)	0.0028 (7)
O5	0.0137 (9)	0.0160 (9)	0.0130 (8)	0.0049 (7)	0.0058 (7)	0.0017 (7)
O10	0.0307 (11)	0.0334 (11)	0.0321 (11)	0.0184 (9)	0.0125 (9)	0.0135 (9)
O12	0.0199 (10)	0.0152 (9)	0.0212 (9)	0.0014 (7)	0.0083 (7)	0.0022 (7)
O2	0.0190 (10)	0.0189 (10)	0.0187 (9)	0.0036 (7)	0.0099 (7)	0.0044 (8)
O11	0.0265 (10)	0.0174 (10)	0.0221 (9)	0.0071 (8)	0.0106 (8)	0.0063 (8)
O7	0.0148 (9)	0.0151 (9)	0.0151 (8)	0.0056 (7)	0.0065 (7)	0.0032 (7)
O4	0.0130 (9)	0.0172 (9)	0.0135 (8)	0.0028 (7)	0.0045 (7)	0.0013 (7)
O1	0.0183 (9)	0.0248 (10)	0.0170 (9)	0.0085 (8)	0.0062 (7)	0.0072 (8)
O14	0.0305 (11)	0.0286 (11)	0.0342 (11)	0.0003 (9)	0.0147 (9)	0.0112 (9)
N3	0.0236 (13)	0.0232 (13)	0.0283 (12)	0.0121 (10)	0.0176 (10)	0.0060 (10)
N1	0.0354 (15)	0.0297 (14)	0.0306 (13)	0.0178 (12)	0.0192 (11)	0.0073 (11)
N2	0.0366 (15)	0.0277 (14)	0.0374 (14)	0.0163 (11)	0.0273 (12)	0.0138 (12)
C9	0.0291 (17)	0.0326 (17)	0.0265 (15)	0.0109 (13)	0.0093 (13)	0.0067 (13)
C3	0.0256 (15)	0.0204 (15)	0.0289 (14)	0.0084 (12)	0.0169 (12)	0.0105 (12)
C15	0.0249 (15)	0.0190 (14)	0.0272 (14)	0.0080 (12)	0.0154 (12)	0.0066 (12)
C7	0.0281 (16)	0.0325 (17)	0.0281 (15)	0.0130 (13)	0.0112 (12)	0.0094 (13)
C17	0.0208 (15)	0.0371 (18)	0.0294 (15)	0.0103 (13)	0.0096 (12)	0.0049 (14)
C2	0.0335 (17)	0.0391 (18)	0.0236 (14)	0.0163 (14)	0.0119 (13)	0.0109 (13)
C14	0.0203 (15)	0.0336 (17)	0.0288 (15)	0.0092 (13)	0.0101 (12)	0.0033 (13)
C10	0.0311 (16)	0.0218 (15)	0.0253 (14)	0.0114 (12)	0.0142 (12)	0.0079 (12)
C6	0.0236 (15)	0.0274 (16)	0.0297 (15)	0.0081 (12)	0.0126 (12)	0.0077 (13)
C8	0.0383 (19)	0.0277 (17)	0.0251 (15)	0.0087 (14)	0.0138 (13)	0.0022 (13)
C4	0.0213 (16)	0.0331 (18)	0.0340 (16)	0.0001 (13)	0.0114 (13)	0.0023 (14)
C16	0.0281 (17)	0.0321 (17)	0.0241 (15)	0.0065 (13)	0.0098 (13)	−0.0027 (13)
C11	0.0320 (18)	0.0323 (18)	0.0323 (16)	0.0081 (14)	0.0097 (14)	−0.0049 (14)
C18	0.0229 (15)	0.0273 (17)	0.0313 (15)	0.0090 (13)	0.0116 (12)	0.0072 (12)
C13	0.0266 (16)	0.0290 (17)	0.0267 (15)	0.0065 (13)	0.0116 (13)	−0.0009 (13)
C5	0.0349 (18)	0.0322 (18)	0.0351 (17)	0.0040 (14)	0.0216 (14)	−0.0009 (14)
C1	0.0306 (17)	0.049 (2)	0.0322 (16)	0.0216 (15)	0.0148 (13)	0.0206 (15)
C12	0.0265 (17)	0.048 (2)	0.0385 (18)	0.0100 (15)	0.0107 (14)	0.0020 (16)

### Geometric parameters (Å, °)

**Table:**

O1W—H19	0.92 (3)	N1—H21	0.8600
O1W—H20	0.92 (3)	N2—C1	1.323 (4)
V1—O13	1.6035 (16)	N2—C5	1.328 (4)
V1—O12	1.7835 (17)	N2—H22	0.8600
V1—O9	1.8657 (17)	C9—C8	1.365 (4)
V1—O4	1.9675 (16)	C9—C10	1.386 (4)
V1—O5	2.0614 (17)	C9—H9	0.9300
V1—O7	2.2568 (15)	C3—C4	1.385 (4)
V4—O6	1.6836 (17)	C3—C2	1.389 (4)
V4—O8	1.6888 (17)	C3—C6	1.459 (3)
V4—O4	1.9082 (15)	C15—C14	1.383 (4)
V4—O5^i^	1.9676 (15)	C15—C16	1.390 (4)
V4—O7	2.0846 (16)	C15—C18	1.469 (3)
V4—O7^i^	2.1459 (16)	C7—C6	1.321 (4)
V5—O3	1.6081 (16)	C7—C10	1.463 (3)
V5—O1	1.7601 (17)	C7—H7	0.9300
V5—O2	1.8682 (17)	C17—C16	1.371 (3)
V5—O4	1.9904 (17)	C17—H17	0.9300
V5—O5	2.0501 (16)	C2—C1	1.374 (4)
V5—O7^i^	2.2376 (15)	C2—H2	0.9300
V2—O14	1.5992 (19)	C14—C13	1.372 (3)
V2—O11	1.8127 (18)	C14—H14	0.9300
V2—O2^i^	1.8635 (16)	C10—C11	1.395 (4)
V2—O12	1.9023 (16)	C6—H6	0.9300
V2—O6	2.0828 (18)	C8—H8	0.9300
V2—O7	2.3437 (17)	C4—C5	1.367 (4)
V3—O10	1.5973 (18)	C4—H4	0.9300
V3—O9	1.8381 (16)	C16—H16	0.9300
V3—O11	1.8461 (18)	C11—C12	1.362 (4)
V3—O1^i^	1.9093 (16)	C11—H11	0.9300
V3—O8^i^	2.0456 (18)	C18—C18^ii^	1.306 (5)
V3—O7	2.3207 (16)	C18—H18	0.9300
N3—C13	1.318 (3)	C13—H13	0.9300
N3—C17	1.331 (3)	C5—H5	0.9300
N3—H23	0.8600	C1—H1	0.9300
N1—C8	1.324 (3)	C12—H12	0.9300
N1—C12	1.339 (4)		
			
H19—O1W—H20	97 (3)	O12—V2—V1	31.33 (5)
O13—V1—O12	104.37 (9)	O6—V2—V1	79.89 (5)
O13—V1—O9	101.50 (8)	O7—V2—V1	46.31 (4)
O12—V1—O9	94.74 (8)	V3—V2—V1	60.491 (14)
O13—V1—O4	100.66 (8)	V4—V2—V1	60.094 (13)
O12—V1—O4	93.50 (7)	O10—V3—O9	101.74 (8)
O9—V1—O4	153.60 (7)	O10—V3—O11	103.94 (9)
O13—V1—O5	98.70 (8)	O9—V3—O11	92.32 (8)
O12—V1—O5	156.10 (7)	O10—V3—O1^i^	102.30 (8)
O9—V1—O5	86.65 (7)	O9—V3—O1^i^	154.79 (8)
O4—V1—O5	75.95 (7)	O11—V3—O1^i^	89.17 (7)
O13—V1—O7	173.33 (8)	O10—V3—O8^i^	99.14 (9)
O12—V1—O7	81.88 (7)	O9—V3—O8^i^	85.43 (7)
O9—V1—O7	80.03 (6)	O11—V3—O8^i^	156.77 (7)
O4—V1—O7	76.37 (6)	O1^i^—V3—O8^i^	83.40 (7)
O5—V1—O7	74.85 (6)	O10—V3—O7	173.91 (9)
O13—V1—V5	89.97 (7)	O9—V3—O7	78.86 (6)
O12—V1—V5	132.01 (6)	O11—V3—O7	82.05 (7)
O9—V1—V5	127.42 (6)	O1^i^—V3—O7	76.43 (6)
O4—V1—V5	38.53 (5)	O8^i^—V3—O7	74.82 (6)
O5—V1—V5	40.77 (5)	O10—V3—V2	136.73 (8)
O7—V1—V5	84.03 (4)	O9—V3—V2	85.19 (5)
O13—V1—V4	136.07 (7)	O11—V3—V2	32.79 (5)
O12—V1—V4	78.71 (5)	O1^i^—V3—V2	82.51 (5)
O9—V1—V4	122.13 (5)	O8^i^—V3—V2	124.07 (5)
O4—V1—V4	36.05 (4)	O7—V3—V2	49.26 (4)
O5—V1—V4	80.37 (4)	O10—V3—V4^i^	129.92 (8)
O7—V1—V4	42.12 (4)	O9—V3—V4^i^	80.78 (6)
V5—V1—V4	60.727 (13)	O11—V3—V4^i^	126.06 (6)
O13—V1—V2	137.78 (7)	O1^i^—V3—V4^i^	78.07 (5)
O12—V1—V2	33.68 (5)	O8^i^—V3—V4^i^	30.78 (5)
O9—V1—V2	83.43 (5)	O7—V3—V4^i^	44.05 (4)
O4—V1—V2	89.73 (5)	V2—V3—V4^i^	93.302 (16)
O5—V1—V2	123.51 (4)	O10—V3—V5^i^	133.07 (7)
O7—V1—V2	48.67 (4)	O9—V3—V5^i^	124.82 (5)
V5—V1—V2	120.397 (16)	O11—V3—V5^i^	81.68 (5)
V4—V1—V2	59.760 (13)	O1^i^—V3—V5^i^	30.77 (5)
O13—V1—V3	134.03 (7)	O8^i^—V3—V5^i^	80.72 (4)
O12—V1—V3	83.67 (6)	O7—V3—V5^i^	45.96 (4)
O9—V1—V3	32.63 (5)	V2—V3—V5^i^	61.047 (14)
O4—V1—V3	124.31 (5)	V4^i^—V3—V5^i^	61.024 (14)
O5—V1—V3	84.82 (5)	O10—V3—V1	134.82 (7)
O7—V1—V3	48.08 (4)	O9—V3—V1	33.18 (5)
V5—V1—V3	118.280 (17)	O11—V3—V1	81.84 (6)
V4—V1—V3	89.836 (15)	O1^i^—V3—V1	122.73 (5)
V2—V1—V3	59.033 (14)	O8^i^—V3—V1	83.76 (5)
O6—V4—O8	106.73 (8)	O7—V3—V1	46.35 (4)
O6—V4—O4	99.21 (8)	V2—V3—V1	60.476 (14)
O8—V4—O4	97.88 (7)	V4^i^—V3—V1	63.607 (14)
O6—V4—O5^i^	96.61 (7)	V5^i^—V3—V1	92.045 (16)
O8—V4—O5^i^	94.96 (7)	V4—O6—V2	110.17 (8)
O4—V4—O5^i^	155.78 (7)	V4—O8—V3^i^	110.92 (8)
O6—V4—O7	88.29 (7)	V3—O9—V1	114.19 (8)
O8—V4—O7	164.73 (8)	V4^i^—O5—V5	106.12 (7)
O4—V4—O7	81.95 (6)	V4^i^—O5—V1	108.17 (7)
O5^i^—V4—O7	80.32 (6)	V5—O5—V1	98.19 (7)
O6—V4—O7^i^	165.98 (7)	V1—O12—V2	114.99 (9)
O8—V4—O7^i^	87.06 (7)	V2^i^—O2—V5	114.19 (8)
O4—V4—O7^i^	80.93 (6)	V2—O11—V3	113.73 (9)
O5^i^—V4—O7^i^	79.31 (6)	V4—O7—V4^i^	102.18 (7)
O7—V4—O7^i^	77.82 (7)	V4—O7—V5^i^	95.92 (6)
O6—V4—V3^i^	144.98 (6)	V4^i^—O7—V5^i^	91.54 (6)
O8—V4—V3^i^	38.30 (6)	V4—O7—V1	91.33 (6)
O4—V4—V3^i^	90.34 (5)	V4^i^—O7—V1	95.64 (6)
O5^i^—V4—V3^i^	87.10 (5)	V5^i^—O7—V1	168.51 (8)
O7—V4—V3^i^	126.54 (5)	V4—O7—V3	170.39 (8)
O7^i^—V4—V3^i^	48.76 (4)	V4^i^—O7—V3	87.20 (6)
O6—V4—V2	39.14 (6)	V5^i^—O7—V3	85.84 (5)
O8—V4—V2	145.85 (6)	V1—O7—V3	85.57 (5)
O4—V4—V2	91.20 (5)	V4—O7—V2	88.55 (6)
O5^i^—V4—V2	89.58 (5)	V4^i^—O7—V2	169.22 (8)
O7—V4—V2	49.16 (5)	V5^i^—O7—V2	86.26 (5)
O7^i^—V4—V2	126.97 (4)	V1—O7—V2	85.02 (5)
V3^i^—V4—V2	175.081 (18)	V3—O7—V2	82.12 (5)
O6—V4—V1	85.84 (6)	V4—O4—V1	106.59 (8)
O8—V4—V1	135.23 (6)	V4—O4—V5	107.36 (8)
O4—V4—V1	37.36 (5)	V1—O4—V5	103.46 (7)
O5^i^—V4—V1	126.81 (5)	V5—O1—V3^i^	115.52 (9)
O7—V4—V1	46.56 (4)	C13—N3—C17	120.5 (2)
O7^i^—V4—V1	85.98 (4)	C13—N3—H23	119.7
V3^i^—V4—V1	119.429 (17)	C17—N3—H23	119.7
V2—V4—V1	60.147 (14)	C8—N1—C12	121.2 (2)
O3—V5—O1	105.70 (8)	C8—N1—H21	119.4
O3—V5—O2	100.00 (8)	C12—N1—H21	119.4
O1—V5—O2	94.80 (8)	C1—N2—C5	120.6 (2)
O3—V5—O4	101.31 (8)	C1—N2—H22	119.7
O1—V5—O4	92.10 (7)	C5—N2—H22	119.7
O2—V5—O4	154.88 (7)	C8—C9—C10	120.1 (3)
O3—V5—O5	97.61 (8)	C8—C9—H9	119.9
O1—V5—O5	155.48 (7)	C10—C9—H9	119.9
O2—V5—O5	88.44 (7)	C4—C3—C2	117.6 (2)
O4—V5—O5	75.71 (7)	C4—C3—C6	119.2 (3)
O3—V5—O7^i^	172.62 (8)	C2—C3—C6	123.2 (3)
O1—V5—O7^i^	81.59 (7)	C14—C15—C16	117.8 (2)
O2—V5—O7^i^	80.23 (6)	C14—C15—C18	118.6 (2)
O4—V5—O7^i^	76.94 (6)	C16—C15—C18	123.6 (2)
O5—V5—O7^i^	75.01 (6)	C6—C7—C10	125.9 (3)
O3—V5—V3^i^	139.16 (7)	C6—C7—H7	117.1
O1—V5—V3^i^	33.71 (5)	C10—C7—H7	117.1
O2—V5—V3^i^	84.31 (5)	N3—C17—C16	121.4 (3)
O4—V5—V3^i^	88.21 (5)	N3—C17—H17	119.3
O5—V5—V3^i^	123.19 (4)	C16—C17—H17	119.3
O7^i^—V5—V3^i^	48.20 (4)	C1—C2—C3	119.2 (3)
O3—V5—V1	89.79 (7)	C1—C2—H2	120.4
O1—V5—V1	130.10 (6)	C3—C2—H2	120.4
O2—V5—V1	129.48 (6)	C13—C14—C15	119.8 (3)
O4—V5—V1	38.01 (5)	C13—C14—H14	120.1
O5—V5—V1	41.04 (5)	C15—C14—H14	120.1
O7^i^—V5—V1	84.49 (4)	C9—C10—C11	117.5 (2)
V3^i^—V5—V1	118.745 (17)	C9—C10—C7	118.9 (3)
O14—V2—O11	105.89 (9)	C11—C10—C7	123.5 (2)
O14—V2—O2^i^	101.51 (8)	C7—C6—C3	125.5 (3)
O11—V2—O2^i^	92.56 (8)	C7—C6—H6	117.3
O14—V2—O12	102.49 (9)	C3—C6—H6	117.3
O11—V2—O12	91.08 (7)	N1—C8—C9	120.7 (3)
O2^i^—V2—O12	153.70 (8)	N1—C8—H8	119.7
O14—V2—O6	99.06 (9)	C9—C8—H8	119.7
O11—V2—O6	155.04 (8)	C5—C4—C3	120.4 (3)
O2^i^—V2—O6	83.03 (7)	C5—C4—H4	119.8
O12—V2—O6	82.74 (7)	C3—C4—H4	119.8
O14—V2—O7	172.02 (9)	C17—C16—C15	119.3 (3)
O11—V2—O7	82.09 (7)	C17—C16—H16	120.4
O2^i^—V2—O7	77.52 (6)	C15—C16—H16	120.4
O12—V2—O7	77.21 (6)	C12—C11—C10	120.0 (3)
O6—V2—O7	72.97 (6)	C12—C11—H11	120.0
O14—V2—V3	139.36 (8)	C10—C11—H11	120.0
O11—V2—V3	33.48 (6)	C18^ii^—C18—C15	125.4 (3)
O2^i^—V2—V3	85.57 (5)	C18^ii^—C18—H18	117.3
O12—V2—V3	83.21 (5)	C15—C18—H18	117.3
O6—V2—V3	121.57 (5)	N3—C13—C14	121.2 (3)
O7—V2—V3	48.62 (4)	N3—C13—H13	119.4
O14—V2—V4	129.74 (8)	C14—C13—H13	119.4
O11—V2—V4	124.36 (6)	N2—C5—C4	120.7 (3)
O2^i^—V2—V4	78.93 (6)	N2—C5—H5	119.7
O12—V2—V4	77.54 (5)	C4—C5—H5	119.7
O6—V2—V4	30.68 (5)	N2—C1—C2	121.6 (3)
O7—V2—V4	42.29 (4)	N2—C1—H1	119.2
V3—V2—V4	90.883 (16)	C2—C1—H1	119.2
O14—V2—V1	133.81 (7)	N1—C12—C11	120.5 (3)
O11—V2—V1	82.33 (5)	N1—C12—H12	119.8
O2^i^—V2—V1	123.82 (5)	C11—C12—H12	119.8
			
O13—V1—V4—O6	−97.82 (11)	O12—V1—V3—O8^i^	159.71 (7)
O12—V1—V4—O6	1.24 (8)	O9—V1—V3—O8^i^	−91.18 (11)
O9—V1—V4—O6	89.76 (9)	O4—V1—V3—O8^i^	69.78 (8)
O4—V1—V4—O6	−111.09 (10)	O5—V1—V3—O8^i^	0.70 (6)
O5—V1—V4—O6	169.60 (7)	O7—V1—V3—O8^i^	74.95 (7)
O7—V1—V4—O6	91.59 (9)	V5—V1—V3—O8^i^	24.88 (5)
V5—V1—V4—O6	−152.17 (6)	V4—V1—V3—O8^i^	81.05 (5)
V2—V1—V4—O6	31.26 (6)	V2—V1—V3—O8^i^	135.20 (5)
V3—V1—V4—O6	84.82 (6)	O13—V1—V3—O7	−171.35 (11)
O13—V1—V4—O8	11.85 (13)	O12—V1—V3—O7	84.76 (8)
O12—V1—V4—O8	110.91 (10)	O9—V1—V3—O7	−166.13 (12)
O9—V1—V4—O8	−160.57 (11)	O4—V1—V3—O7	−5.17 (8)
O4—V1—V4—O8	−1.42 (11)	O5—V1—V3—O7	−74.25 (7)
O5—V1—V4—O8	−80.73 (10)	V5—V1—V3—O7	−50.07 (6)
O7—V1—V4—O8	−158.74 (11)	V4—V1—V3—O7	6.10 (6)
V5—V1—V4—O8	−42.50 (9)	V2—V1—V3—O7	60.25 (6)
V2—V1—V4—O8	140.93 (9)	O13—V1—V3—V2	128.40 (10)
V3—V1—V4—O8	−165.51 (9)	O12—V1—V3—V2	24.51 (5)
O13—V1—V4—O4	13.27 (12)	O9—V1—V3—V2	133.62 (10)
O12—V1—V4—O4	112.33 (10)	O4—V1—V3—V2	−65.42 (6)
O9—V1—V4—O4	−159.15 (11)	O5—V1—V3—V2	−134.50 (4)
O5—V1—V4—O4	−79.31 (9)	O7—V1—V3—V2	−60.25 (6)
O7—V1—V4—O4	−157.32 (11)	V5—V1—V3—V2	−110.319 (19)
V5—V1—V4—O4	−41.08 (8)	V4—V1—V3—V2	−54.152 (15)
V2—V1—V4—O4	142.35 (8)	O13—V1—V3—V4^i^	−120.81 (10)
V3—V1—V4—O4	−164.09 (8)	O12—V1—V3—V4^i^	135.30 (5)
O13—V1—V4—O5^i^	167.03 (11)	O9—V1—V3—V4^i^	−115.59 (10)
O12—V1—V4—O5^i^	−93.91 (8)	O4—V1—V3—V4^i^	45.37 (6)
O9—V1—V4—O5^i^	−5.39 (9)	O5—V1—V3—V4^i^	−23.71 (4)
O4—V1—V4—O5^i^	153.76 (10)	O7—V1—V3—V4^i^	50.54 (6)
O5—V1—V4—O5^i^	74.45 (9)	V5—V1—V3—V4^i^	0.470 (16)
O7—V1—V4—O5^i^	−3.56 (9)	V4—V1—V3—V4^i^	56.637 (16)
V5—V1—V4—O5^i^	112.68 (6)	V2—V1—V3—V4^i^	110.789 (16)
V2—V1—V4—O5^i^	−63.89 (6)	O13—V1—V3—V5^i^	−176.84 (10)
V3—V1—V4—O5^i^	−10.33 (7)	O12—V1—V3—V5^i^	79.28 (5)
O13—V1—V4—O7	170.59 (12)	O9—V1—V3—V5^i^	−171.61 (10)
O12—V1—V4—O7	−90.35 (8)	O4—V1—V3—V5^i^	−10.65 (6)
O9—V1—V4—O7	−1.83 (9)	O5—V1—V3—V5^i^	−79.73 (4)
O4—V1—V4—O7	157.32 (11)	O7—V1—V3—V5^i^	−5.48 (6)
O5—V1—V4—O7	78.01 (8)	V5—V1—V3—V5^i^	−55.55 (2)
V5—V1—V4—O7	116.24 (6)	V4—V1—V3—V5^i^	0.614 (16)
V2—V1—V4—O7	−60.33 (6)	V2—V1—V3—V5^i^	54.766 (15)
V3—V1—V4—O7	−6.77 (6)	O8—V4—O6—V2	178.36 (7)
O13—V1—V4—O7^i^	93.58 (11)	O4—V4—O6—V2	−80.47 (9)
O12—V1—V4—O7^i^	−167.36 (7)	O5^i^—V4—O6—V2	81.13 (8)
O9—V1—V4—O7^i^	−78.84 (8)	O7—V4—O6—V2	1.09 (8)
O4—V1—V4—O7^i^	80.31 (9)	O7^i^—V4—O6—V2	9.0 (3)
O5—V1—V4—O7^i^	1.00 (6)	V3^i^—V4—O6—V2	175.54 (3)
O7—V1—V4—O7^i^	−77.01 (8)	V1—V4—O6—V2	−45.47 (6)
V5—V1—V4—O7^i^	39.23 (4)	O14—V2—O6—V4	179.34 (9)
V2—V1—V4—O7^i^	−137.34 (4)	O11—V2—O6—V4	1.0 (2)
V3—V1—V4—O7^i^	−83.78 (4)	O2^i^—V2—O6—V4	−80.05 (9)
O13—V1—V4—V3^i^	56.56 (10)	O12—V2—O6—V4	77.78 (9)
O12—V1—V4—V3^i^	155.62 (6)	O7—V2—O6—V4	−1.01 (7)
O9—V1—V4—V3^i^	−115.86 (7)	V3—V2—O6—V4	0.37 (10)
O4—V1—V4—V3^i^	43.29 (8)	V1—V2—O6—V4	46.21 (7)
O5—V1—V4—V3^i^	−36.02 (5)	O6—V4—O8—V3^i^	−177.39 (7)
O7—V1—V4—V3^i^	−114.03 (6)	O4—V4—O8—V3^i^	80.47 (9)
V5—V1—V4—V3^i^	2.210 (17)	O5^i^—V4—O8—V3^i^	−78.95 (8)
V2—V1—V4—V3^i^	−174.36 (2)	O7—V4—O8—V3^i^	−7.8 (3)
V3—V1—V4—V3^i^	−120.797 (17)	O7^i^—V4—O8—V3^i^	0.05 (8)
O13—V1—V4—V2	−129.08 (10)	V2—V4—O8—V3^i^	−175.54 (3)
O12—V1—V4—V2	−30.02 (6)	V1—V4—O8—V3^i^	81.34 (10)
O9—V1—V4—V2	58.51 (7)	O10—V3—O9—V1	−176.00 (10)
O4—V1—V4—V2	−142.35 (8)	O11—V3—O9—V1	−71.24 (10)
O5—V1—V4—V2	138.34 (5)	O1^i^—V3—O9—V1	21.7 (2)
O7—V1—V4—V2	60.33 (6)	O8^i^—V3—O9—V1	85.59 (10)
V5—V1—V4—V2	176.571 (17)	O7—V3—O9—V1	10.18 (9)
V3—V1—V4—V2	53.564 (15)	V2—V3—O9—V1	−39.21 (8)
O13—V1—V5—O3	1.55 (9)	V4^i^—V3—O9—V1	54.93 (8)
O12—V1—V5—O3	111.02 (10)	V5^i^—V3—O9—V1	10.23 (12)
O9—V1—V5—O3	−102.96 (9)	O13—V1—O9—V3	176.17 (10)
O4—V1—V5—O3	108.86 (10)	O12—V1—O9—V3	70.45 (10)
O5—V1—V5—O3	−101.88 (9)	O4—V1—O9—V3	−37.3 (2)
O7—V1—V5—O3	−175.55 (7)	O5—V1—O9—V3	−85.63 (9)
V4—V1—V5—O3	147.23 (7)	O7—V1—O9—V3	−10.44 (9)
V2—V1—V5—O3	150.67 (6)	V5—V1—O9—V3	−84.92 (10)
V3—V1—V5—O3	−140.54 (6)	V4—V1—O9—V3	−9.19 (12)
O13—V1—V5—O1	−108.99 (10)	V2—V1—O9—V3	38.67 (8)
O12—V1—V5—O1	0.49 (10)	O3—V5—O5—V4^i^	−167.47 (9)
O9—V1—V5—O1	146.50 (9)	O1—V5—O5—V4^i^	30.6 (2)
O4—V1—V5—O1	−1.67 (9)	O2—V5—O5—V4^i^	−67.59 (8)
O5—V1—V5—O1	147.58 (10)	O4—V5—O5—V4^i^	92.73 (8)
O7—V1—V5—O1	73.92 (8)	O7^i^—V5—O5—V4^i^	12.71 (7)
V4—V1—V5—O1	36.70 (7)	V3^i^—V5—O5—V4^i^	14.57 (9)
V2—V1—V5—O1	40.13 (7)	V1—V5—O5—V4^i^	111.69 (9)
V3—V1—V5—O1	108.92 (7)	O3—V5—O5—V1	80.85 (9)
O13—V1—V5—O2	104.36 (9)	O1—V5—O5—V1	−81.12 (19)
O12—V1—V5—O2	−146.16 (10)	O2—V5—O5—V1	−179.28 (6)
O9—V1—V5—O2	−0.14 (9)	O4—V5—O5—V1	−18.95 (6)
O4—V1—V5—O2	−148.32 (10)	O7^i^—V5—O5—V1	−98.97 (7)
O5—V1—V5—O2	0.94 (8)	V3^i^—V5—O5—V1	−97.12 (6)
O7—V1—V5—O2	−72.73 (8)	O13—V1—O5—V4^i^	170.24 (9)
V4—V1—V5—O2	−109.95 (6)	O12—V1—O5—V4^i^	−25.0 (2)
V2—V1—V5—O2	−106.51 (6)	O9—V1—O5—V4^i^	69.11 (8)
V3—V1—V5—O2	−37.73 (7)	O4—V1—O5—V4^i^	−90.86 (8)
O13—V1—V5—O4	−107.31 (10)	O7—V1—O5—V4^i^	−11.46 (7)
O12—V1—V5—O4	2.16 (10)	V5—V1—O5—V4^i^	−110.03 (9)
O9—V1—V5—O4	148.18 (9)	V4—V1—O5—V4^i^	−54.27 (6)
O5—V1—V5—O4	149.26 (10)	V2—V1—O5—V4^i^	−10.74 (9)
O7—V1—V5—O4	75.59 (8)	V3—V1—O5—V4^i^	36.44 (6)
V4—V1—V5—O4	38.37 (7)	O13—V1—O5—V5	−79.73 (9)
V2—V1—V5—O4	41.81 (7)	O12—V1—O5—V5	84.99 (19)
V3—V1—V5—O4	110.60 (7)	O9—V1—O5—V5	179.14 (7)
O13—V1—V5—O5	103.43 (9)	O4—V1—O5—V5	19.16 (6)
O12—V1—V5—O5	−147.09 (10)	O7—V1—O5—V5	98.57 (6)
O9—V1—V5—O5	−1.08 (8)	V4—V1—O5—V5	55.75 (5)
O4—V1—V5—O5	−149.26 (10)	V2—V1—O5—V5	99.29 (6)
O7—V1—V5—O5	−73.67 (7)	V3—V1—O5—V5	146.47 (5)
V4—V1—V5—O5	−110.89 (6)	O13—V1—O12—V2	−173.81 (10)
V2—V1—V5—O5	−107.45 (6)	O9—V1—O12—V2	−70.64 (10)
V3—V1—V5—O5	−38.66 (6)	O4—V1—O12—V2	84.25 (10)
O13—V1—V5—O7^i^	176.88 (8)	O5—V1—O12—V2	21.8 (2)
O12—V1—V5—O7^i^	−73.64 (8)	O7—V1—O12—V2	8.56 (9)
O9—V1—V5—O7^i^	72.37 (7)	V5—V1—O12—V2	82.90 (11)
O4—V1—V5—O7^i^	−75.80 (8)	V4—V1—O12—V2	51.20 (8)
O5—V1—V5—O7^i^	73.45 (7)	V3—V1—O12—V2	−39.90 (8)
O7—V1—V5—O7^i^	−0.21 (8)	O14—V2—O12—V1	179.81 (10)
V4—V1—V5—O7^i^	−37.43 (4)	O11—V2—O12—V1	73.27 (10)
V2—V1—V5—O7^i^	−34.00 (4)	O2^i^—V2—O12—V1	−24.7 (2)
V3—V1—V5—O7^i^	34.79 (4)	O6—V2—O12—V1	−82.47 (10)
O13—V1—V5—V3^i^	−147.87 (7)	O7—V2—O12—V1	−8.37 (9)
O12—V1—V5—V3^i^	−38.39 (8)	V3—V2—O12—V1	40.66 (8)
O9—V1—V5—V3^i^	107.62 (6)	V4—V2—O12—V1	−51.75 (8)
O4—V1—V5—V3^i^	−40.55 (7)	O3—V5—O2—V2^i^	−175.44 (10)
O5—V1—V5—V3^i^	108.71 (6)	O1—V5—O2—V2^i^	−68.54 (10)
O7—V1—V5—V3^i^	35.04 (4)	O4—V5—O2—V2^i^	36.9 (2)
V4—V1—V5—V3^i^	−2.180 (17)	O5—V5—O2—V2^i^	87.11 (9)
V2—V1—V5—V3^i^	1.25 (3)	O7^i^—V5—O2—V2^i^	12.05 (9)
V3—V1—V5—V3^i^	70.04 (3)	V3^i^—V5—O2—V2^i^	−36.46 (8)
O6—V4—V2—O14	−0.85 (12)	V1—V5—O2—V2^i^	86.49 (9)
O8—V4—V2—O14	−3.65 (14)	O14—V2—O11—V3	−179.26 (9)
O4—V4—V2—O14	102.33 (10)	O2^i^—V2—O11—V3	78.01 (10)
O5^i^—V4—V2—O14	−101.88 (10)	O12—V2—O11—V3	−75.94 (10)
O7—V4—V2—O14	−179.41 (10)	O6—V2—O11—V3	−0.9 (2)
O7^i^—V4—V2—O14	−178.13 (10)	O7—V2—O11—V3	0.98 (8)
V1—V4—V2—O14	124.09 (9)	V4—V2—O11—V3	−0.33 (12)
O6—V4—V2—O11	−179.50 (11)	V1—V2—O11—V3	−45.78 (7)
O8—V4—V2—O11	177.70 (12)	O10—V3—O11—V2	−179.88 (9)
O4—V4—V2—O11	−76.33 (8)	O9—V3—O11—V2	77.42 (10)
O5^i^—V4—V2—O11	79.46 (8)	O1^i^—V3—O11—V2	−77.41 (10)
O7—V4—V2—O11	1.94 (8)	O8^i^—V3—O11—V2	−6.4 (2)
O7^i^—V4—V2—O11	3.21 (9)	O7—V3—O11—V2	−0.99 (8)
V1—V4—V2—O11	−54.57 (7)	V4^i^—V3—O11—V2	−2.86 (12)
O6—V4—V2—O2^i^	95.00 (10)	V5^i^—V3—O11—V2	−47.45 (8)
O8—V4—V2—O2^i^	92.20 (11)	V1—V3—O11—V2	45.85 (8)
O4—V4—V2—O2^i^	−161.82 (7)	O6—V4—O7—V4^i^	178.05 (7)
O5^i^—V4—V2—O2^i^	−6.03 (7)	O8—V4—O7—V4^i^	8.0 (3)
O7—V4—V2—O2^i^	−83.56 (7)	O4—V4—O7—V4^i^	−82.40 (7)
O7^i^—V4—V2—O2^i^	−82.28 (7)	O5^i^—V4—O7—V4^i^	81.04 (7)
V1—V4—V2—O2^i^	−140.06 (5)	O7^i^—V4—O7—V4^i^	0.0
O6—V4—V2—O12	−96.83 (10)	V3^i^—V4—O7—V4^i^	2.01 (8)
O8—V4—V2—O12	−99.63 (11)	V2—V4—O7—V4^i^	178.96 (8)
O4—V4—V2—O12	6.34 (7)	V1—V4—O7—V4^i^	−96.06 (7)
O5^i^—V4—V2—O12	162.13 (7)	O6—V4—O7—V5^i^	85.18 (7)
O7—V4—V2—O12	84.61 (7)	O8—V4—O7—V5^i^	−84.8 (3)
O7^i^—V4—V2—O12	85.88 (7)	O4—V4—O7—V5^i^	−175.26 (7)
V1—V4—V2—O12	28.10 (5)	O5^i^—V4—O7—V5^i^	−11.82 (6)
O8—V4—V2—O6	−2.80 (12)	O7^i^—V4—O7—V5^i^	−92.86 (7)
O4—V4—V2—O6	103.17 (10)	V3^i^—V4—O7—V5^i^	−90.85 (6)
O5^i^—V4—V2—O6	−101.04 (10)	V2—V4—O7—V5^i^	86.09 (6)
O7—V4—V2—O6	−178.56 (10)	V1—V4—O7—V5^i^	171.07 (9)
O7^i^—V4—V2—O6	−177.29 (10)	O6—V4—O7—V1	−85.89 (7)
V1—V4—V2—O6	124.93 (9)	O8—V4—O7—V1	104.1 (3)
O6—V4—V2—O7	178.56 (10)	O4—V4—O7—V1	13.67 (6)
O8—V4—V2—O7	175.76 (11)	O5^i^—V4—O7—V1	177.11 (7)
O4—V4—V2—O7	−78.26 (7)	O7^i^—V4—O7—V1	96.06 (7)
O5^i^—V4—V2—O7	77.53 (7)	V3^i^—V4—O7—V1	98.08 (5)
O7^i^—V4—V2—O7	1.28 (10)	V2—V4—O7—V1	−84.98 (5)
V1—V4—V2—O7	−56.50 (5)	O6—V4—O7—V2	−0.91 (6)
O6—V4—V2—V3	−179.68 (9)	O8—V4—O7—V2	−170.9 (2)
O8—V4—V2—V3	177.52 (10)	O4—V4—O7—V2	98.65 (6)
O4—V4—V2—V3	−76.51 (5)	O5^i^—V4—O7—V2	−97.91 (6)
O5^i^—V4—V2—V3	79.28 (5)	O7^i^—V4—O7—V2	−178.96 (8)
O7—V4—V2—V3	1.75 (5)	V3^i^—V4—O7—V2	−176.95 (2)
O7^i^—V4—V2—V3	3.03 (5)	V1—V4—O7—V2	84.98 (5)
V1—V4—V2—V3	−54.750 (15)	O12—V1—O7—V4	82.12 (7)
O6—V4—V2—V1	−124.93 (9)	O9—V1—O7—V4	178.43 (7)
O8—V4—V2—V1	−127.73 (10)	O4—V1—O7—V4	−13.50 (6)
O4—V4—V2—V1	−21.76 (5)	O5—V1—O7—V4	−92.36 (7)
O5^i^—V4—V2—V1	134.03 (5)	V5—V1—O7—V4	−51.88 (5)
O7—V4—V2—V1	56.50 (5)	V2—V1—O7—V4	88.43 (6)
O7^i^—V4—V2—V1	57.78 (5)	V3—V1—O7—V4	170.89 (8)
O13—V1—V2—O14	8.69 (15)	O12—V1—O7—V4^i^	−175.50 (7)
O12—V1—V2—O14	−0.26 (14)	O9—V1—O7—V4^i^	−79.19 (7)
O9—V1—V2—O14	108.58 (12)	O4—V1—O7—V4^i^	88.88 (7)
O4—V1—V2—O14	−96.98 (11)	O5—V1—O7—V4^i^	10.01 (6)
O5—V1—V2—O14	−169.86 (11)	V5—V1—O7—V4^i^	50.50 (5)
O7—V1—V2—O14	−168.94 (12)	V4—V1—O7—V4^i^	102.38 (7)
V5—V1—V2—O14	−121.52 (10)	V2—V1—O7—V4^i^	−169.19 (8)
V4—V1—V2—O14	−118.05 (10)	V3—V1—O7—V4^i^	−86.74 (6)
V3—V1—V2—O14	131.71 (10)	O12—V1—O7—V5^i^	−47.1 (4)
O13—V1—V2—O11	−96.00 (12)	O9—V1—O7—V5^i^	49.2 (4)
O12—V1—V2—O11	−104.95 (11)	O4—V1—O7—V5^i^	−142.7 (4)
O9—V1—V2—O11	3.88 (7)	O5—V1—O7—V5^i^	138.4 (4)
O4—V1—V2—O11	158.33 (7)	V5—V1—O7—V5^i^	178.9 (4)
O5—V1—V2—O11	85.44 (8)	V4—V1—O7—V5^i^	−129.2 (4)
O7—V1—V2—O11	86.36 (8)	V2—V1—O7—V5^i^	−40.8 (4)
V5—V1—V2—O11	133.79 (6)	V3—V1—O7—V5^i^	41.7 (4)
V4—V1—V2—O11	137.26 (6)	O12—V1—O7—V3	−88.77 (7)
V3—V1—V2—O11	27.02 (6)	O9—V1—O7—V3	7.54 (7)
O13—V1—V2—O2^i^	176.06 (12)	O4—V1—O7—V3	175.61 (7)
O12—V1—V2—O2^i^	167.11 (12)	O5—V1—O7—V3	96.75 (6)
O9—V1—V2—O2^i^	−84.06 (8)	V5—V1—O7—V3	137.24 (4)
O4—V1—V2—O2^i^	70.39 (8)	V4—V1—O7—V3	−170.89 (8)
O5—V1—V2—O2^i^	−2.50 (9)	V2—V1—O7—V3	−82.46 (5)
O7—V1—V2—O2^i^	−1.58 (8)	O12—V1—O7—V2	−6.31 (7)
V5—V1—V2—O2^i^	45.85 (7)	O9—V1—O7—V2	90.00 (7)
V4—V1—V2—O2^i^	49.32 (7)	O4—V1—O7—V2	−101.93 (6)
V3—V1—V2—O2^i^	−60.92 (7)	O5—V1—O7—V2	179.20 (6)
O13—V1—V2—O12	8.95 (14)	V5—V1—O7—V2	−140.31 (4)
O9—V1—V2—O12	108.84 (11)	V4—V1—O7—V2	−88.43 (6)
O4—V1—V2—O12	−96.72 (11)	V3—V1—O7—V2	82.46 (5)
O5—V1—V2—O12	−169.61 (12)	O9—V3—O7—V4^i^	88.20 (7)
O7—V1—V2—O12	−168.68 (12)	O11—V3—O7—V4^i^	−177.83 (7)
V5—V1—V2—O12	−121.26 (10)	O1^i^—V3—O7—V4^i^	−86.77 (7)
V4—V1—V2—O12	−117.79 (10)	O8^i^—V3—O7—V4^i^	−0.03 (5)
V3—V1—V2—O12	131.97 (10)	V2—V3—O7—V4^i^	−178.54 (7)
O13—V1—V2—O6	101.59 (11)	V5^i^—V3—O7—V4^i^	−91.75 (6)
O12—V1—V2—O6	92.64 (11)	V1—V3—O7—V4^i^	95.89 (6)
O9—V1—V2—O6	−158.52 (7)	O9—V3—O7—V5^i^	179.95 (7)
O4—V1—V2—O6	−4.08 (6)	O11—V3—O7—V5^i^	−86.09 (7)
O5—V1—V2—O6	−76.96 (7)	O1^i^—V3—O7—V5^i^	4.98 (6)
O7—V1—V2—O6	−76.04 (7)	O8^i^—V3—O7—V5^i^	91.71 (6)
V5—V1—V2—O6	−28.61 (5)	V2—V3—O7—V5^i^	−86.80 (5)
V4—V1—V2—O6	−25.15 (5)	V4^i^—V3—O7—V5^i^	91.75 (6)
V3—V1—V2—O6	−135.39 (5)	V1—V3—O7—V5^i^	−172.37 (8)
O13—V1—V2—O7	177.63 (12)	O9—V3—O7—V1	−7.69 (7)
O12—V1—V2—O7	168.68 (12)	O11—V3—O7—V1	86.28 (7)
O9—V1—V2—O7	−82.48 (7)	O1^i^—V3—O7—V1	177.34 (7)
O4—V1—V2—O7	71.96 (7)	O8^i^—V3—O7—V1	−95.92 (6)
O5—V1—V2—O7	−0.92 (7)	V2—V3—O7—V1	85.57 (5)
V5—V1—V2—O7	47.43 (5)	V4^i^—V3—O7—V1	−95.89 (6)
V4—V1—V2—O7	50.89 (5)	V5^i^—V3—O7—V1	172.37 (8)
V3—V1—V2—O7	−59.35 (5)	O9—V3—O7—V2	−93.26 (6)
O13—V1—V2—V3	−123.02 (11)	O11—V3—O7—V2	0.71 (6)
O12—V1—V2—V3	−131.97 (10)	O1^i^—V3—O7—V2	91.78 (6)
O9—V1—V2—V3	−23.13 (5)	O8^i^—V3—O7—V2	178.51 (6)
O4—V1—V2—V3	131.31 (5)	V4^i^—V3—O7—V2	178.54 (7)
O5—V1—V2—V3	58.42 (6)	V5^i^—V3—O7—V2	86.80 (5)
O7—V1—V2—V3	59.35 (5)	V1—V3—O7—V2	−85.57 (5)
V5—V1—V2—V3	106.77 (2)	O11—V2—O7—V4	−178.39 (7)
V4—V1—V2—V3	110.240 (17)	O2^i^—V2—O7—V4	87.20 (7)
O13—V1—V2—V4	126.74 (11)	O12—V2—O7—V4	−85.45 (7)
O12—V1—V2—V4	117.79 (10)	O6—V2—O7—V4	0.77 (5)
O9—V1—V2—V4	−133.38 (5)	V3—V2—O7—V4	−177.66 (7)
O4—V1—V2—V4	21.07 (5)	V1—V2—O7—V4	−91.46 (6)
O5—V1—V2—V4	−51.82 (6)	O11—V2—O7—V4^i^	7.1 (4)
O7—V1—V2—V4	−50.89 (5)	O2^i^—V2—O7—V4^i^	−87.3 (4)
V5—V1—V2—V4	−3.468 (17)	O12—V2—O7—V4^i^	100.0 (4)
V3—V1—V2—V4	−110.240 (17)	O6—V2—O7—V4^i^	−173.8 (4)
O14—V2—V3—O10	1.27 (15)	V3—V2—O7—V4^i^	7.8 (4)
O11—V2—V3—O10	0.17 (13)	V4—V2—O7—V4^i^	−174.5 (4)
O2^i^—V2—V3—O10	−101.28 (11)	V1—V2—O7—V4^i^	94.0 (4)
O12—V2—V3—O10	102.55 (11)	O11—V2—O7—V5^i^	85.58 (7)
O6—V2—V3—O10	179.70 (11)	O2^i^—V2—O7—V5^i^	−8.83 (6)
O7—V2—V3—O10	−178.53 (11)	O12—V2—O7—V5^i^	178.52 (7)
V4—V2—V3—O10	179.89 (10)	O6—V2—O7—V5^i^	−95.26 (6)
V1—V2—V3—O10	125.46 (10)	V3—V2—O7—V5^i^	86.31 (5)
O14—V2—V3—O9	−100.77 (12)	V4—V2—O7—V5^i^	−96.03 (6)
O11—V2—V3—O9	−101.87 (11)	V1—V2—O7—V5^i^	172.51 (7)
O2^i^—V2—V3—O9	156.69 (8)	O11—V2—O7—V1	−86.93 (6)
O12—V2—V3—O9	0.52 (8)	O2^i^—V2—O7—V1	178.66 (7)
O6—V2—V3—O9	77.67 (8)	O12—V2—O7—V1	6.00 (6)
O7—V2—V3—O9	79.43 (7)	O6—V2—O7—V1	92.23 (6)
V4—V2—V3—O9	77.86 (6)	V3—V2—O7—V1	−86.20 (5)
V1—V2—V3—O9	23.43 (6)	V4—V2—O7—V1	91.46 (6)
O14—V2—V3—O11	1.10 (14)	O11—V2—O7—V3	−0.72 (6)
O2^i^—V2—V3—O11	−101.44 (11)	O2^i^—V2—O7—V3	−95.14 (6)
O12—V2—V3—O11	102.39 (11)	O12—V2—O7—V3	92.21 (6)
O6—V2—V3—O11	179.54 (11)	O6—V2—O7—V3	178.43 (6)
O7—V2—V3—O11	−178.70 (11)	V4—V2—O7—V3	177.66 (7)
V4—V2—V3—O11	179.73 (10)	V1—V2—O7—V3	86.20 (5)
V1—V2—V3—O11	125.30 (10)	O6—V4—O4—V1	70.51 (9)
O14—V2—V3—O1^i^	101.28 (12)	O8—V4—O4—V1	178.99 (8)
O11—V2—V3—O1^i^	100.19 (11)	O5^i^—V4—O4—V1	−59.7 (2)
O2^i^—V2—V3—O1^i^	−1.26 (8)	O7—V4—O4—V1	−16.42 (8)
O12—V2—V3—O1^i^	−157.43 (8)	O7^i^—V4—O4—V1	−95.29 (8)
O6—V2—V3—O1^i^	−80.28 (8)	V3^i^—V4—O4—V1	−143.33 (7)
O7—V2—V3—O1^i^	−78.52 (7)	V2—V4—O4—V1	32.00 (7)
V4—V2—V3—O1^i^	−80.09 (6)	O6—V4—O4—V5	−179.14 (8)
V1—V2—V3—O1^i^	−134.52 (6)	O8—V4—O4—V5	−70.66 (9)
O14—V2—V3—O8^i^	178.06 (12)	O5^i^—V4—O4—V5	50.7 (2)
O11—V2—V3—O8^i^	176.96 (11)	O7—V4—O4—V5	93.92 (8)
O2^i^—V2—V3—O8^i^	75.52 (8)	O7^i^—V4—O4—V5	15.06 (7)
O12—V2—V3—O8^i^	−80.65 (8)	V3^i^—V4—O4—V5	−32.98 (7)
O6—V2—V3—O8^i^	−3.50 (8)	V2—V4—O4—V5	142.35 (6)
O7—V2—V3—O8^i^	−1.74 (7)	V1—V4—O4—V5	110.35 (10)
V4—V2—V3—O8^i^	−3.31 (6)	O13—V1—O4—V4	−170.68 (9)
V1—V2—V3—O8^i^	−57.74 (6)	O12—V1—O4—V4	−65.34 (9)
O14—V2—V3—O7	179.80 (12)	O9—V1—O4—V4	42.7 (2)
O11—V2—V3—O7	178.70 (11)	O5—V1—O4—V4	92.92 (8)
O2^i^—V2—V3—O7	77.26 (7)	O7—V1—O4—V4	15.43 (7)
O12—V2—V3—O7	−78.91 (7)	V5—V1—O4—V4	113.05 (10)
O6—V2—V3—O7	−1.76 (7)	V2—V1—O4—V4	−31.85 (7)
V4—V2—V3—O7	−1.57 (5)	V3—V1—O4—V4	19.38 (10)
V1—V2—V3—O7	−56.00 (5)	O13—V1—O4—V5	76.28 (9)
O14—V2—V3—V4^i^	178.79 (11)	O12—V1—O4—V5	−178.39 (7)
O11—V2—V3—V4^i^	177.69 (10)	O9—V1—O4—V5	−70.36 (18)
O2^i^—V2—V3—V4^i^	76.24 (6)	O5—V1—O4—V5	−20.13 (6)
O12—V2—V3—V4^i^	−79.93 (5)	O7—V1—O4—V5	−97.62 (7)
O6—V2—V3—V4^i^	−2.78 (6)	V4—V1—O4—V5	−113.05 (10)
O7—V2—V3—V4^i^	−1.01 (5)	V2—V1—O4—V5	−144.90 (6)
V4—V2—V3—V4^i^	−2.588 (18)	V3—V1—O4—V5	−93.66 (6)
V1—V2—V3—V4^i^	−57.018 (14)	O3—V5—O4—V4	172.70 (8)
O14—V2—V3—V5^i^	124.69 (11)	O1—V5—O4—V4	66.23 (9)
O11—V2—V3—V5^i^	123.59 (10)	O2—V5—O4—V4	−39.8 (2)
O2^i^—V2—V3—V5^i^	22.15 (5)	O5—V5—O4—V4	−92.23 (8)
O12—V2—V3—V5^i^	−134.02 (6)	O7^i^—V5—O4—V4	−14.63 (7)
O6—V2—V3—V5^i^	−56.87 (5)	V3^i^—V5—O4—V4	32.74 (7)
O7—V2—V3—V5^i^	−55.11 (5)	V1—V5—O4—V4	−112.49 (10)
V4—V2—V3—V5^i^	−56.681 (14)	O3—V5—O4—V1	−74.81 (9)
V1—V2—V3—V5^i^	−111.111 (17)	O1—V5—O4—V1	178.72 (7)
O14—V2—V3—V1	−124.20 (11)	O2—V5—O4—V1	72.72 (17)
O11—V2—V3—V1	−125.30 (10)	O5—V5—O4—V1	20.26 (6)
O2^i^—V2—V3—V1	133.26 (6)	O7^i^—V5—O4—V1	97.86 (7)
O12—V2—V3—V1	−22.91 (5)	V3^i^—V5—O4—V1	145.23 (6)
O6—V2—V3—V1	54.24 (5)	O3—V5—O1—V3^i^	174.03 (10)
O7—V2—V3—V1	56.00 (5)	O2—V5—O1—V3^i^	72.23 (10)
V4—V2—V3—V1	54.430 (14)	O4—V5—O1—V3^i^	−83.60 (10)
O13—V1—V3—O10	0.30 (15)	O5—V5—O1—V3^i^	−24.6 (2)
O12—V1—V3—O10	−103.59 (12)	O7^i^—V5—O1—V3^i^	−7.14 (9)
O9—V1—V3—O10	5.53 (14)	V1—V5—O1—V3^i^	−82.57 (11)
O4—V1—V3—O10	166.49 (12)	C13—N3—C17—C16	−0.7 (5)
O5—V1—V3—O10	97.40 (12)	C4—C3—C2—C1	−1.0 (4)
O7—V1—V3—O10	171.65 (12)	C6—C3—C2—C1	177.3 (3)
V5—V1—V3—O10	121.58 (11)	C16—C15—C14—C13	−0.6 (5)
V4—V1—V3—O10	177.75 (11)	C18—C15—C14—C13	179.3 (3)
V2—V1—V3—O10	−128.10 (11)	C8—C9—C10—C11	1.7 (4)
O13—V1—V3—O9	−5.23 (13)	C8—C9—C10—C7	−176.6 (3)
O12—V1—V3—O9	−109.11 (12)	C6—C7—C10—C9	170.6 (3)
O4—V1—V3—O9	160.96 (12)	C6—C7—C10—C11	−7.6 (5)
O5—V1—V3—O9	91.88 (11)	C10—C7—C6—C3	−178.3 (3)
O7—V1—V3—O9	166.13 (12)	C4—C3—C6—C7	175.9 (3)
V5—V1—V3—O9	116.06 (10)	C2—C3—C6—C7	−2.3 (5)
V4—V1—V3—O9	172.22 (10)	C12—N1—C8—C9	−0.6 (5)
V2—V1—V3—O9	−133.62 (10)	C10—C9—C8—N1	−0.8 (5)
O13—V1—V3—O11	101.87 (11)	C2—C3—C4—C5	0.3 (5)
O12—V1—V3—O11	−2.02 (7)	C6—C3—C4—C5	−178.0 (3)
O9—V1—V3—O11	107.10 (12)	N3—C17—C16—C15	0.6 (5)
O4—V1—V3—O11	−91.94 (8)	C14—C15—C16—C17	0.1 (5)
O5—V1—V3—O11	−161.03 (7)	C18—C15—C16—C17	−179.8 (3)
O7—V1—V3—O11	−86.77 (8)	C9—C10—C11—C12	−1.2 (5)
V5—V1—V3—O11	−136.84 (5)	C7—C10—C11—C12	177.0 (3)
V4—V1—V3—O11	−80.68 (5)	C14—C15—C18—C18^ii^	−179.5 (4)
V2—V1—V3—O11	−26.52 (5)	C16—C15—C18—C18^ii^	0.5 (6)
O13—V1—V3—O1^i^	−174.42 (11)	C17—N3—C13—C14	0.1 (5)
O12—V1—V3—O1^i^	81.69 (8)	C15—C14—C13—N3	0.6 (5)
O9—V1—V3—O1^i^	−169.20 (12)	C1—N2—C5—C4	−1.8 (5)
O4—V1—V3—O1^i^	−8.24 (9)	C3—C4—C5—N2	1.1 (5)
O5—V1—V3—O1^i^	−77.32 (8)	C5—N2—C1—C2	1.1 (5)
O7—V1—V3—O1^i^	−3.07 (8)	C3—C2—C1—N2	0.3 (5)
V5—V1—V3—O1^i^	−53.14 (7)	C8—N1—C12—C11	1.2 (5)
V4—V1—V3—O1^i^	3.03 (7)	C8—N1—C12—C11	1.2 (5)
V2—V1—V3—O1^i^	57.18 (7)	C10—C11—C12—N1	−0.2 (5)
O13—V1—V3—O8^i^	−96.40 (10)		

Symmetry codes: (i) −*x*+1, −*y*, −*z*; (ii) −*x*, −*y*+1, −*z*+1.

### Hydrogen-bond geometry (Å, °)

**Table:**

*D*—H···*A*	*D*—H	H···*A*	*D*···*A*	*D*—H···*A*
O1w—H19···O11^iii^	0.92 (3)	2.01 (4)	2.919 (3)	168 (4)
O1w—H20···O12	0.92 (3)	2.15 (3)	3.028 (3)	160 (3)
N1—H21···O9	0.86	1.88	2.721 (3)	164
N2—H22···O2^ii^	0.86	1.75	2.568 (3)	159
N3—H23···O5	0.86	1.71	2.564 (3)	172
C2—H2···O1^iv^	0.93	2.50	3.126 (4)	124
C4—H4···O1^v^	0.93	2.32	3.107 (4)	143
C8—H8···O10	0.93	2.57	3.162 (4)	122
C11—H11···O3^v^	0.93	2.33	3.254 (4)	173
C12—H12···O1w	0.93	2.57	3.230 (4)	128
C14—H14···O6^vi^	0.93	2.54	3.202 (3)	128
C14—H14···O12^vi^	0.93	2.39	3.293 (3)	162
C16—H16···O13^v^	0.93	2.58	3.401 (4)	147
C17—H17···O13	0.93	2.60	3.187 (4)	122

Symmetry codes: (iii) −*x*+1, −*y*+1, −*z*; (ii) −*x*, −*y*+1, −*z*+1; (iv) *x*−1, *y*+1, *z*; (v) −*x*+1, −*y*+1, −*z*+1; (vi) *x*−1, *y*, *z*.

## References

[bb1] Altomare, A., Cascarano, G., Giacovazzo, C. & Guagliardi, A. (1993). *J. Appl. Cryst.* **26**, 343–350.

[bb2] Blatov, A. V. (2006). *IUCr CompComm. Newslett.* **7**, 4–6.

[bb3] Fernández de Luis, R., Mesa, J. L., Urtiaga, M. K., Rojo, T. & Arriortua, M. I. (2009*a*). *Eur. J. Inorg. Chem.* pp. 4786–4794.

[bb4] Fernández de Luis, R., Urtiaga, M. K., Mesa, J. L., Rojo, T. & Arriortua, M. I. (2009*b*). *J. Alloys Compd*, **480**, 54–56.

[bb5] Khan, M. I., Nome, R. C., Ayesh, S., Golub, V. O., O’Connor, C. J. & Doedens, R. J. (2004). *Chem. Mater.* **16**, 5273–5279.

[bb6] Oxford Diffraction (2008). *CrysAlis CCD and *CrysAlis RED** Oxford Diffraction Ltd, Abingdon, England.

[bb7] Pope, M. T. & Müller, A. (1991). *Angew. Chem. Int. Ed. Engl.* **30**, 34–48.

[bb8] Pope, M. T. & Müller, A. (1994). *Polyoxometalates: From Platonic Solids to Anti-retroviral Activity* Drodrecht, The Netherlands: Kluwer.

[bb9] Rhule, J. T., Hill, C. L., Judd, D. A. & Schinazi, R. F. (1998). *Chem. Rev.* **98**, 327–358. 10.1021/cr960396q 11851509

[bb10] Sheldrick, G. M. (2008). *Acta Cryst.* A**64**, 112–122. 10.1107/S0108767307043930 18156677

[bb11] Spek, A. L. (2009). *Acta Cryst.* D**65**, 148–155. 10.1107/S090744490804362X PMC2631630 19171970

[bb12] Steiner, T. (2002). *Angew. Chem. Int. Ed.* **41**, 48–76.

[bb13] Zavalij, P. Y. & Whittingham, M. S. (1999). *Acta Cryst.* B**55**, 627–663. 10.1107/s0108768199004000 10927405

[bb14] Zheng, L.-M., Wang, X., Wang, Y. & Jacobson, A. J. (2001). *J. Mater. Chem.* **11**, 1100–1105.

